# No correlation among expressed emotion, anxiety, stress and weight loss in patients with overweight and obesity

**DOI:** 10.29219/fnr.v63.3522

**Published:** 2019-10-08

**Authors:** Carla Gramaglia, Eleonora Gattoni, Camilla Vecchi, Elisa Di Tullio, Giampaolo Biroli, Federico D’Andrea, Sergio Riso, Maria Rosaria Gualano, Marco Marchetti, Marco Sarchiapone, Roberta Siliquini, Patrizia Zeppegno

**Affiliations:** 1 Institute of Psychiatry, Università degli Studi del Piemonte Orientale, Novara, Italy; 2 S.C. Psichiatria, Azienda Ospedaliero Universitaria Maggiore della Carità, Novara, Italy; 3 S.C. Dietetica e Nutrizione, Azienda Ospedaliero Universitaria Maggiore della Carità, Novara, Italy; 4 Department of Public Health, University of Torino, Torino, Italy; 5 Department of Bioscience and Territory, University of Molise, Campobasso, Italy; 6 Department of Medical and Health Sciences, University of Molise, Campobasso, Italy

**Keywords:** expressed emotion, anxiety, stress, obesity, overweight

## Abstract

**Background:**

The onset of some types of obesity may correlate with specific familial relational patterns, and expressed emotion (EE), the family life’s ‘emotional temperature’, may play a role in obesity treatment compliance and outcome.

**Objective:**

The aim of this study is to address the current gap in the literature about EE and obesity, assessing EE in a sample of patients with overweight or obesity and their relatives. A further objective is to assess patients’ weight loss, patients’ and relatives’ anxiety, perceived stress and their possible correlation with EE and diet compliance.

**Design:**

A total of 220 patients with overweight or obesity and 126 relatives were recruited; their socio-demographic and clinical features were collected; and Level of Expressed Emotion Scale (LEE), State-Trait Anxiety Inventory 1 and 2 (STAI-Y1 and STAI-Y2) and Paykel Scale of Stressful Life Events were administered.

**Results:**

Patients’ baseline body mass index (BMI) was negatively correlated with educational level, but we failed to find any correlation between BMI and the other variables assessed. We found a positive correlation between EE median and stressful life events, as well as between median EE and state and trait anxiety.

**Conclusions:**

Our results seem to suggest that other factors than the psychological ones we investigated may play a role in treatment adherence and outcome in patients with overweight and obesity.

## Popular scientific summary

- Obesity is a complex condition with medical, social and psychological implications, that affects all age and socioeconomic groups.
- The identification of possible correlates of obesity might offer targets for interventions.
- We studied the relation between family life’s “emotional temperature” and body weight in a sample of overweight and obese patients and their relatives.
- We found that body weight was related with schooling, furthermore the emotional temperature is related to stress and anxiety.

Overweight and obesity may cause severe physical disabilities and psychological problems, and drastically increase the risk of developing several non-communicable diseases, including cardiovascular disease, cancer and diabetes (1), with relevant consequences and impact on patients’ well-being and public health. Hence, in this population of patients, it is important to study factors influencing diet adherence and weight-loss maintenance (2).

Familial behaviours, emotional environment and quality of family relationships have raised the interest of clinical researchers because of their relationship with the expression, development, maintenance and treatment response of both psychiatric and physical disorders (3). To capture this concept, it developed the expressed emotion (EE) construct (4), which describes changeable aspects ‘of ongoing family interactions’ (5), also called the family life’s ‘blood pressure’ (6), or ‘emotional temperature’ (7).

The onset of some types of obesity may correlate with specific familial relational patterns, and EE may play a role in obesity treatment compliance and outcome. Bruch suggested that the main reason for early-onset obesity could be the mothers’ difficulty to discriminate between their children’s emotional needs, hunger or actual need for food (8). Differences in the recognition of facial expression were found between mothers of children with severe early-onset obesity and control mothers, suggesting that emotional decoding difficulties could play a role in the development of obesity (9). Critical and negative comments on adolescents’ weight and shape may have an impact on self-esteem and emotional development and may contribute to the onset of excessive weight and shape concern, which is a risk factor for the development of eating disordered behaviours (10). Overeating has been hypothesized to be a consequence of the suppression of negative emotions, and to serve to (temporarily) repair negative mood (11). Furthermore, it is well known that emotional states are key elements in eating disorders (EDs) generally with anger and aggressiveness playing an important role (12).

Nonetheless, to our knowledge, only a few and quite outdated studies have addressed EE in patients with obesity. A study involving a sample of 30 key relatives of 30 patients with severe obesity found that patients living with a high EE relative were less likely to comply with treatment (rated according to weight loss or gain at a 5-month follow-up) than those living with a low EE relative (13). Another study performed in a small sample of US women found EE correctly predicting weight maintenance for 78.5% of the cases (14). On the other hand, more research has been performed about the relation between EE and EDs, highlighting a correlation between eating attitudes, weight and body shape and the emotional response and tolerance of influential person (15), as well as an association of caregiver eating-related messages (encouraging to eat or to restrict food intake, body dissatisfaction and eating disturbances) (16).

Besides a possible, and still rather unexplored, relation with EE, the literature has supported a bidirectional association between obesity and other psychological correlates such as mood and anxiety disorders, weight stigma and perceived stress (17–19). Furthermore, a recent literature review highlights that weight stigma is consistently associated with mental health status, anxiety, perceived stress, antisocial behaviour, substance use and medication non-adherence (19). The aim of this study is to address the current gap in the literature about EE and obesity, assessing the EE construct in a sample of patients with overweight or obesity and their relatives. A further objective is to assess patients’ weight loss, patients’ and relatives’ anxiety, perceived stress and their possible correlation with EE and diet compliance. Moreover, we aimed to analyse whether there were differences between patients whose relatives agreed to participate in the study and those who did not and between patients who continued follow-up and patients who dropped out. We decided to compare these two groups in order to test whether differences in levels of EE, anxiety and stress levels could explain relatives’ and patients’ unwillingness to participate in the study and to adhere to medical checks, respectively.

## Methods

Patients with overweight or obesity were recruited from the 1st November 2011 to the 1st May 2015 at the Nutrition Ward of the Azienda Ospedaliera Universitaria ‘Maggiore della Carità’ Hospital, Novara, Italy. Inclusion criteria for patients were the following: (1) diagnosis of overweight or obesity [body mass index (BMI) ≥ 25], (2) age ≥ 18 years, (3) no comorbidity with major psychiatric disorders (schizophrenia, mood disorders), (4) willingness to provide informed written consent and (5) proper understanding of the Italian language. No exclusion criteria were adopted for relatives (who also included cohabiting partners). Patients and relatives were asked to provide an informed written consent.

A total of 220 patients and 126 relatives were recruited, their socio-demographic features were collected and they were asked to complete the self-administered questionnaires described below. Patients’ baseline assessment (T0) also included BMI measure and gathering of information about comorbidities (e.g. cardiovascular disease, diabetes, liver steatosis and sleep apnoea syndrome). According to BMI, patients were categorized into four groups: overweight (25–29.9 Kg/m^2^), obesity I (30–34.9 Kg/m^2^), obesity II (35–39.9 Kg/m^2^) and obesity III (≥40 Kg/m^2^) (20). Patients’ BMI was measured 3 months after baseline (T1), and at the study endpoint (6 months follow-up, T2). Weight loss was assumed as a marker of diet compliance, as in other previous studies (13).

Patients’ and relatives’ assessment included the following tests:

### Level of Expressed Emotion Scale

The Level of Expressed Emotion Scale (LEE) (21) is a 60-item, self-report measure that assesses the emotional environment in the patient’s most important relationship. It has been developed as a reliable alternative to overcome the limits of the Camberwell Family Interview (CFI), the EE gold standard measure, whose practical application in clinical settings is hindered by its time-consuming administration and rating (22).

The LEE consists of four subscales: intrusiveness, emotional response, attitude towards illness and tolerance and expectation. Items are rated on a true–false format, and the scale generates a score for the overall level of EE (median) as well as a score for each of the four subscales. Two versions of the LEE scale were used: the patient version, asking patients to evaluate the relationship with their closest relative (i.e. the person with whom they live, enrolled in this study); and the relative version, requiring the closest person to evaluate his or her relationship with the patient (22).

### State-Trait Anxiety Inventory 1 and 2

The State-Trait Anxiety Inventory (STAI) is a widely used measure of trait and state anxiety (23). The State Anxiety Scale, State-Trait Anxiety Inventory 1 (STAI-Y1) (S-Anxiety), evaluates the current state of anxiety, asking how respondents feel ‘right now’, using items that measure subjective feelings of apprehension, tension, nervousness, worry and activation/arousal of the autonomic nervous system. The Trait Anxiety Scale, State-Trait Anxiety Inventory 2 (STAI-Y2) (T-Anxiety), evaluates relatively stable aspects of ‘anxiety proneness’, including general states of calmness, confidence and security (24).

### Paykel scale of stressful life events

This is a 61-item instrument which covers a comprehensive range of recent life events occurred during the 6 months before the assessment (25). The interview categorizes life events of moderate to severe degree in 10 groups as follows: employment, education, financial status, somatic health, loss (death of close relatives), living place, relationship, criminality, family and social problems and marital problems (26).

### Statistical analysis

Descriptive statistics, including means, medians, standard deviations (SD) and range (Min – Max), were calculated for continuous variables. Categorical variables were expressed as frequencies. Differences between continuous variables and categorical variables were assessed using *t*-test and chi-square test, respectively. Correlations between variables (scales) were explored using Pearson’s correlation coefficient. Questionnaire scores and socio-demographic variables were compared between patients whose relatives took part to the assessment and patients whose relatives refused to participate; furthermore, comparisons were also performed between the patients lost at follow-up and those who completed the study and between patients and relatives. Longitudinal differences in the study variables were tested by using repeated measures analysis of variance to find statistically significant differences between means of variables values at each time, controlling variability between subjects. Results of *P*-value at Greenhouse–Geisser test were considered. Boxplots were used to show BMI trend. Data were analysed with SPSS 22.0 software for Windows, and level of significance was set at *P* ≤ 0.05 (27).

## Results

### Socio-demographic and clinical features and questionnaire scores in the whole sample (patients and relatives)

Of the 220 patients with overweight and obesity recruited, 147 were females (66.8%) and 73 were males (33.2%); their mean age was 45.34 ± 15.38 years (age range 18–76 years). Data could be collected for 126 relatives, while 94 (42.7%) refused participation in the study. In the relatives’ group, 66 were females (52.4%) and 60 males (47.6%); their mean age was 49.10 ± 14.72 (age range 19–75 years). Patients’ and relatives’ socio-demographic features are summarized in Table 1.

**Table 1:** Patients’ and relatives’ socio-demographic features

		Patients (*n* = 220)		Relatives (*n* = 126)	
		*N*	%	*N*	%
Level of education	Primary school	33	15	19	15.1
	Junior high school	90	40.9	53	42.1
	High school	82	37.3	40	31.7
	Degree	15	6.8	14	11.1
Marital status	Married	141	64.1	89	70.6
	Single	58	26.4	21	16.7
	Divorced or widowed	21	9.5	16	12.7
Employment	Employed	109	49.5	63	50
	Unemployed	111	50.5	63	50

LEE, STAI-Y1, STAI-Y2 and Paykel scale scores and the *P*-values of the *t*-test performed to compare the questionnaire scores in the two groups of patients and relatives are reported in Table 2.

**Table 2:** Patients’ and relatives’ test results

	Patients (*n* = 220) (mean ± SD)	Relatives (*n* = 126) (mean ± SD)	*P*
Level of Expressed Emotion Scale (LEE)	Expressed emotion (EE) median	3.90 ± 2.938	3.26 ± 2.417	0.116
	Intrusiveness	5.48 ± 3.806	4.90 ± 3.344	0.202
	Emotional response	4.31 ± 3.665	3.73 ± 3.191	0.385
	Attitude toward disease	2.53 ± 2.709	2.02 ± 1.894	0.175
	Tolerance and expectation	3.90 ± 3.097	3.34 ± 2.618	0.162
State anxiety (STAI Y1)		41.45 ± 13.063	37.08 ± 11.057	**0.042**
Trait anxiety (STAI Y2)		43.12 ± 12.553	39.7 ± 10.852	**0.008**
Life events (Paykel)		38.63 ± 31.639	30.15 ± 34.624	0.171

P ≤ 0.05.

At baseline 17.7% (*n* = 39) of patients were overweight, while 35% (*n* = 77), 18.2% (*n* = 40) and 15.5% (*n* = 34) of patients were classified as obesity classes I–III, respectively. For 13.6% (*n* = 30) patients, BMI was missing. Comorbidities were found in 95 patients (43.2%) and were more frequent in female (*P* < 0.02) and older patients (*P* < 0.001), and in patients with higher BMI (*P* = 0.037). Marital status seemed to be related with these comorbidities (*P* < 0.02): married patients, patients living with the partner and widowed or divorced patients had more comorbidities linked to obesity.

**Fig. 1:**
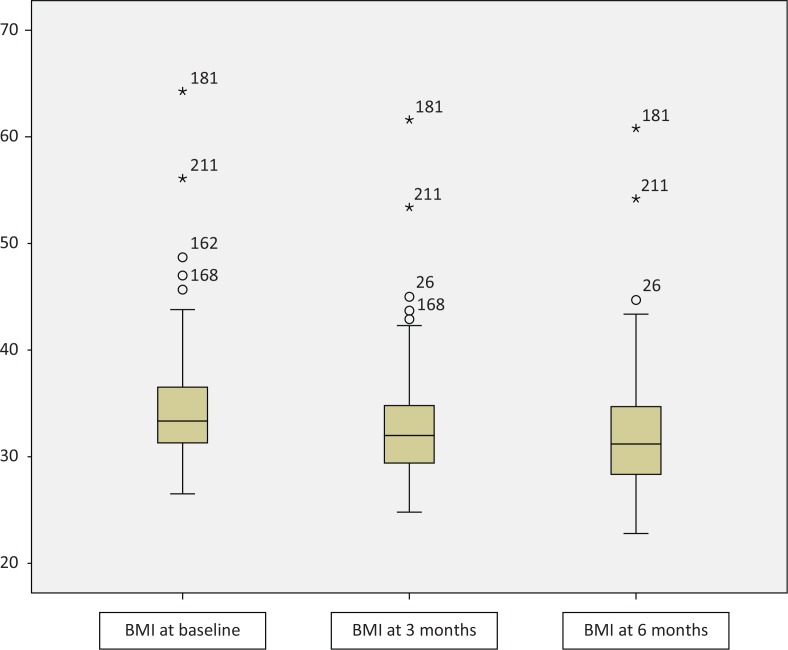
Box-plot showing BMI trend over time, *P* < 0.001.

Patients’ dropout rate was 47.72 and 59.55% at T1 and T2, respectively. Only 89 (40.45%) patients completed the planned 6-months follow-up, and a statistically significant decrease in BMI from T0 to T2 was found (see Fig. 1).

### Correlation analysis – patients’ sample

Patients’ baseline BMI was negatively correlated with educational level (*P* = 0.006), but we failed to find any correlation between BMI and the other variables assessed (sex, age, employment status, family history of psychiatric disorder, comorbidities, EE median, stressful life events and state and trait anxiety). Furthermore, Pearson’s correlations yielded the following results (only statistically significant ones are described). A positive correlation was found between the EE median and female gender (*P* = 0.043), while a negative one was found between EE median and level of education (*P* = 0.031). A positive correlation was found between the EE median and stressful life events (Paykel scale, *P* < 0.001), and between EE median and state and trait anxiety (STAI-Y1, *P* < 0.001; STAI-Y2, *P* < 0.001). A negative correlation was found between trait anxiety and age (*P* = 0.001), while a positive correlation emerged between female gender and both trait anxiety (*P* < 0.001) and state anxiety (*P* = 0.001). The Paykel scale showed a negative correlation with age (*P* = 0.001), a positive one with employment status (*P* = 0.006) and family history of psychiatric disorder (*P* = 0.02).

### Correlation analysis: relatives’ sample

We found a positive correlation between EE median and stressful life events (Paykel scale, *P* = 0.035), as well as between median EE and state and trait anxiety (STAI-Y1, *P* = 0.001; STAI-Y2, *P* = 0.000), respectively.

A negative correlation was found between trait anxiety and age (*P* = 0.037) and a positive correlation between trait anxiety and female gender (*P* = 0.006).

### Patients with relatives versus patients without relatives

No differences in any of the variables assessed (sex, age, employment status, family history of psychiatric disorder, BMI, comorbidities, EE median, stressful life events and state and trait anxiety) emerged from the comparison between patients whose relatives took part in the study and those whose relatives were not available to do so.

### Patients with follow-up data versus dropouts

The comparison between the patients who completed the study and those lost at follow-up after baseline (sex, age, employment status, family history of psychiatric disorder, BMI, comorbidities, EE median, stressful life events and state and trait anxiety were compared) highlighted that the latter were less frequently males (41.94 vs. 23.96%; *P* = 0.0106), younger (mean age 47.16 vs. 42.98; *P* =0.045) and less likely to have obesity-related comorbidities (51.61 vs. 32.29%; *P* = 0.004).

## Discussion

Overweight and obesity are associated with poor quality of life, with a possible impact on psychological and social well-being (28). Although the physical and health implications of overweight are widely acknowledged, and research has shown that overweight individuals show high levels of depression, high perceived stress and low self-esteem, overweight individuals are often stereotyped as being happy-go-lucky and carefree (17–19, 29). This stereotyped image of patients with obesity could lead to difficulties perceiving overweight and obesity as actual health problems, and we cannot exclude that this might have played a role on the poor treatment adherence (59.55 % dropout from T0 to T2) and difficult involvement of relatives (42.7 % refused to participate) in the current study.

The patients’ baseline sample was mainly composed by women (66.8 %), consistent with clinical practice and literature data showing that women are more willing to seek help and to express and disclose their sufferance (30, 31).

### Correlation analyses

In the patients’ sample, no correlation was found between BMI and any of the other study variables. Only one significant, negative correlation was found between baseline BMI and educational level. In other words, in our sample of patients, a higher level of education correlated with a lower baseline BMI. Epidemiological data available in our country suggest that overweight and obesity may be linked to a lower level of education (41% patients with overweight and 23% patients with obesity have a low educational level or are illiterate) (32).

We found a positive correlation between EE median score (LEE), Paykel Scale of Stressful Life Events, STAI-Y1 and STAI-Y2 scores in both the patients’ and relatives’ group, suggesting higher overall levels of EE in individuals experiencing more stressful life events and higher degrees of state and trait anxiety. Several studies involving patients affected by different disorders (e.g. patients with myocardial infarction, EDs) have supported a positive correlation between EE and levels of anxiety (33, 34). EE levels have been reported to be influenced by stressful life events and perceived stress as well in research studies of psychotic patients (35).

Moreover, in the patients’ sample only, further correlations of EE median were found, namely a positive one with female gender and a negative one with educational level. Some studies highlighted that the perception of emotions is influenced by gender. Indeed, women have been reported to be more negatively affected by criticism in the context of relationships than men (36).

Regarding the STAI-Y scales, we found a positive correlation between trait anxiety and female gender, and a negative one between trait anxiety and age, both in the patients’ and relatives’ groups. The first finding is consistent with the literature, suggesting that women are twice as likely to have an anxiety disorder (37), while the second is supported by reports about a lower prevalence of anxiety and some anxiety-related disorders (e.g. phobias, panic and obsessive-compulsive disorders) in older patients than in younger ones (38).

Furthermore, in the patients’ sample only, a positive correlation was found between state anxiety and gender. Higher state anxiety among females was also described in other samples (39). It is likely that this result was found only among patients because they were recruited while they were waiting for the medical examination, a situation that may lead to feelings of apprehension, tension, nervousness and worry.

### Patients with relatives versus patients without relatives

While some studies suggested that perceived EE could have an impact on patients’ choice to involve their relatives in treatment (29, 40), our results conversely failed to find any differences between the level of median EE between patients whose relatives accepted to participate to the study and those patients whose relatives did not.

### Patients with follow-up data versus dropouts

Surprisingly, even if our results about the baseline sample composition are consistent with the literature data highlighting men’s difficulty in seeking help, in our study, patients lost at follow-up were less likely male. This suggests that after getting engaged into treatment, male patients may be more willing than female ones to go on with it.

Furthermore, patients who drop out after the first diet visit were younger and less frequently affected by obesity-related disorder. Younger patients with no comorbidities may be less likely to perceive their obesity as an actual disorder. On the other hand, concurrent diseases, such as hypertension, diabetes, myocardial infarction, angina pectoris, dyslipidaemia, sleep apnoea and liver steatosis, may motivate the patient to continue with diet visits, as suggested also by recent research follow-up studies in patients with obesity undergoing bariatric surgery (41).

## Conclusions

Although perceived EE is a valuable predictor of clinical outcome in psychiatric and community samples (42), to our knowledge, this is one of the few available studies focused on obesity, overweight and EE in adult patients (13, 14). Therefore, we believe that one of the strengths of our research is addressing the existing gap in the literature in this specific field of research. Moreover, the exclusion of patients with comorbid major psychiatric disorders, whose correlation with EE is widely acknowledged, allowed us to avoid biases studying the actual correlations among overweight/obesity and the other variables we assessed. Lastly, the dual approach we adopted, including objective measures of relatives’ actual EE and measures of patients’ perceptions of their relatives’ emotional stance, likely provides a more thorough and balanced perspective in the assessment of the patient–caregiver dyad (43).

Some limitations should be underscored as well. Even if these data likely depend on difficulties perceiving overweight and obesity as proper diseases, regrettably, only 57.3% of relatives were willing to participate in the study, and patients’ dropout rate during the 6-months follow-up after baseline was 59.55%. Regarding the study design, we did not gather clinical information (e.g. BMI) in the relatives’ group, which could have been useful to understand overall eating- and weight-related family behaviours; moreover, we did not include a sample of healthy controls.

Summing up, the high dropout rate we registered in our sample suggests that overweight and obesity may not be perceived as actual diseases, and that their possible impact on overall physical and psychological health status (2) is likely still highly underestimated by affected people and their relatives.

We failed to find any correlation among EE, anxiety, stressful life events and BMI in our sample of patients with overweight or obesity, even though we found correlations among EE, anxiety and stress. As far as EE levels are concerned, it should be pointed out that according to our research procedure, we recruited patients who decided to undergo a diet visit, representing a selected sample, which is certainly not fully representative of all the population with obesity and overweight in the community. Moreover, we cannot exclude that the involvement of those families who refused to participate to the current study might have yielded different results, since higher EE may be correlated with less compliance. Some studies in the field of addiction underlined that higher family conflict and poor-family cohesion are linked to poorer treatment outcomes and a greater level of relapses; conversely, less EE and greater warmth are associated with lower risk of relapses (44). Moreover, Eray and colleagues showed that perceived EE can hinder treatment compliance in a sample of diabetic adolescents (45).

On the other hand, notwithstanding all the limitations described above, our results seem to suggest that other factors than the psychological ones we investigated may play a role in treatment adherence and outcome in patients with overweight and obesity.

## Conflict of interest and funding

The authors declared no conflict of interest. The authors have not received any funding or benefits from industry or elsewhere to conduct this study.
